# Cost-effectiveness analysis of implementing polygenic risk score in a workplace cardiovascular disease prevention program

**DOI:** 10.3389/fpubh.2023.1139496

**Published:** 2023-07-11

**Authors:** Deo Mujwara, Jen Kintzle, Paolo Di Domenico, George B. Busby, Giordano Bottà

**Affiliations:** Allelica, Inc., New York, NY, United States

**Keywords:** cost-effectiveness, polygenic risk score (PRS), cardiovasccular risk factors, workplace setting, prevention

## Abstract

**Background:**

Polygenic risk score for coronary artery disease (CAD-PRS) improves precision in assessing the risk of cardiovascular diseases and is cost-effective in preventing cardiovascular diseases in a health system and may be cost-effective in other settings and prevention programs such as workplace cardiovascular prevention programs. Workplaces provide a conducitve environment for cardiovascular prevention interventions, but the cost-effectiveness of CAD-PRS in a workplace setting remains unknown. This study examined the cost-effectiveness of integrating CAD-PRS in a workplace cardiovascular disease prevention program compared to the standard cardiovascular workplace program without CAD-PRS and no-workplace prevention program.

**Methods:**

We developed a cohort simulation model to project health benefits (quality-adjusted life years gained) and costs over a period of 5 years in a cohort of employees with a mean age of 50 years. The model health states reflected the risk of disease (coronary artery disease and ischemic stroke) and statin prevention therapy side effects (diabetes, hemorrhagic stroke, and myopathy). We considered medical and lost productivity costs. Data were obtained from the literature, and the analysis was performed from a self-insured employer perspective with future costs and quality-adjusted life years discounted at 3% annually. Uncertainty in model parameter inputs was assessed using deterministic and probabilistic sensitivity analyses. Three programs were compared: (1) a workplace cardiovascular program that integrated CAD-PRS with the pooled cohort equation—a standard of care for assessing the risk of cardiovascular diseases (CardioriskSCORE); (2) a workplace cardiovascular prevention program without CAD-PRS (Standard-WHP); and (3) no-workplace health program (No-WHP). The main outcomes were total costs (US $2019), incremental costs, incremental quality-adjusted life years, and incremental cost-effectiveness ratio.

**Results:**

CardioriskSCORE lowered employer costs ($53 and $575) and improved employee quality-adjusted life years (0.001 and 0.005) per employee screened compared to Standard-WHP and No-WHP, respectively. The effectiveness of statin prevention therapy, employees' baseline cardiovascular risk, the proportion of employees that enrolled in the program, and statin adherence had the largest effect size on the incremental net monetary benefit. However, despite the variation in parameter input values, base case results remained robust.

**Conclusion:**

Polygenic testing in a workplace cardiovascular prevention program improves employees' quality of life and simultaneously lowers health costs and productivity monetary loss for employers.

## Introduction

Workplaces provide a convenient environment for cardiovascular disease prevention interventions. However, standard workplace healthcare programs (WHPs) only screen for traditional risk factors (e.g., age, blood pressure, cholesterol) for cardiovascular diseases (1) without accounting for genetic risk. There is strong evidence that a substantial proportion of coronary artery disease (CAD) is attributable to genetic factors (2) and that genetics modify the risk conferred by traditional risk factors (3). As such, employees at high risk of CAD due to genetics remain invisible to current risk assessments, thereby missing opportunities for preventative disease interventions to be initiated that can improve employees' health and wellbeing and lower future healthcare costs.

A polygenic risk score (PRS) is a number that indicates an individual's risk of disease, estimated using large clinical biobanks by integrating multiple risk variant alleles for an individual weighted by their effect on disease risk. Adding PRS for coronary artery disease (CAD-PRS) in current risk assessment models improves precision in determining the risk of CAD, identifying an additional 4% of the primary prevention population at risk of CAD who would otherwise remain unidentified using only traditional risk factors (2). CAD-PRS is also an independent predictor of CAD (2, 4) and has been recommended (2) and shown to be cost-effective and cost-saving in a health system when integrated into the current risk assessment models as an additional risk-enhancing factor (5).

There is a growing interest in offering genetic testing to employees, but its cost-effectiveness has not been fully examined (6, 7). Furthermore, WHPs that include genetic testing offer only monogenic testing (6), which identifies fewer individuals at high risk compared to polygenic testing (8). The objective of this study was to examine the cost-effectiveness of CAD-PRS in a workplace cardiovascular disease prevention program compared to a standard workplace cardiovascular prevention program and a workplace without any prevention program.

## Materials and methods

### Study population

The study population consisted of a cohort of individuals with a mean age of 50 years, representing the average age of employees between 40 and 75 years in the United States (85), which is also the recommended age for cardiovascular disease prevention interventions (9). The cohort excluded individuals with pre-existing conditions, such as diabetes (10) and CAD/stroke (11), as those are not classified among the primary prevention population for cardiovascular diseases (9).

### Strategies

We compared three strategies: (i) standard workplace program using a pooled cohort equation (PCE) for assessing the 10-year risk of CAD based on traditional risk factors (Standard-WHP). The risk is stratified into four categories: low (< 5%), borderline (5% to < 7.5%), intermediate (≥7.5% to < 20%), and high (≥20%). (ii) CAD-PRS integrated with the PCE (CardioriskSCORE). (iii) No-workplace health program (No-WHP). In CardioriskSCORE, employees first assess their PCE 10-year risk and then self-administer a non-invasive oral DNA test, which is analyzed in a Clinical Laboratory Improvement Amendments (CLIA) certified and College of American Pathologists (CAP) accredited laboratory to calculate individual CAD-PRS. Within 3 weeks, employees receive confidential and personalized results for their disease risk based on the combination of CAD-PRS and PCE risk. Those at high risk are recommended to initiate statin preventive therapy through their primary care physician or cardiologist. Employees have access to a Health Assistant Mobile App to track their health status and modifiable risk factors over time and are supplied with personalized recommendations to maintain a healthy diet and lifestyle.

### Model structure

**Figure 1:**
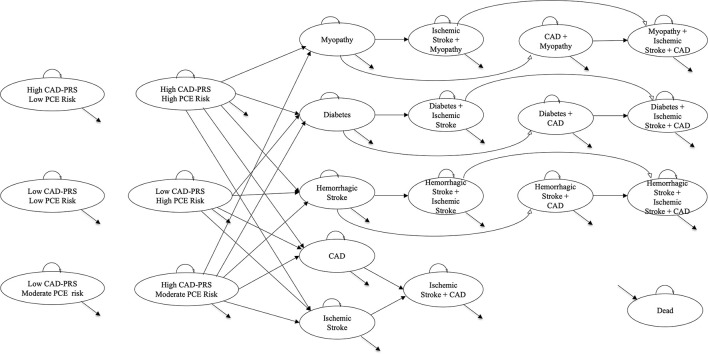
Markov model schematic. This figure shows the Markov model schematic with 22 health states representing the risk of CAD, health outcomes, statin side effects, and death: 6 of 22 health states represented the risk of CAD based on PCE (low PCE risk, moderate PCE risk, and high PCE risk) and CAD-PRS (low CAD-PRS = bottom 80% of the PRS distribution and high CAD-PRS = top 20% of the PRS distribution); 3 of 22 health states represented statin side effects (myopathy, diabetes, and hemorrhagic stroke); 2 of 22 health states represented the primary health outcomes (CAD and ischemic stroke); 10 of 22 health states represented comorbidities of statin side effects and/or primary health outcomes; finally, 1/22 health states represented death. Outcomes were examined for the proportion of the cohort that was eligible for prevention intervention (i.e., high CAD-PRS with high/moderate PCE risk; low CAD-PRS and high PCE risk). In the CardioriskSCORE strategy, all the cohorts eligible for prevention intervention initiated statin preventive therapy to reduce the risk of CAD and ischemic stroke, while for the Standard-WHP strategy, only a proportion with high PCE risk initiated prevention therapy, and none in the No-WHP. CAD, coronary artery disease; CAD-PRS, polygenic risk score for coronary artery disease; PCE, pooled cohort equation.

We developed a cohort simulation model (Figure 1) in TreeAge Pro Software 2021 to project costs and quality-adjusted life years (QALYs) among employees in a workplace setting. We used a simulation model as no patient-level data (real-world data) were available to examine the economic impact and health benefits of implementing CAD-PRS in a workplace setting. The model had an annual cycle with a total of 22 health states reflecting the risk of CAD stratified by PCE and CAD-PRS, side effects of statin prevention therapy (diabetes, hemorrhagic stroke, and myopathy), health outcomes (CAD and ischemic stroke), and mortality.

We assumed the cohort to be disease-free at the start of the model and individuals classified as eligible for the prevention intervention could develop CAD and ischemic stroke over the set time horizon. Fractions of the cohort that was eligible and adherent to statin prevention therapy had a reduced risk of developing CAD and ischemic stroke but were also at risk of developing statin side effects. We accounted for both disease-specific and age-adjusted natural mortality, and utility values and costs were applied to respective health states to project QALYs and costs. The model was validated by comparing the lifetime expectancy in the disease-free cohort to that of the general US population and the number of adverse event outcomes in prior published work (5). This manuscript followed the 2022 CHEERS checklist (Supplementary Table S1).

### Parameter inputs

Parameter inputs (Table 1) used in the model were derived from the literature. The initial distribution of the cohort came from a large (*N* = 47,108 persons) multi-centric multi-ancestry study conducted in the United States with the cohort classified by PCE 10-year risk [low (41%), moderate (36%), and high risk (23%)] and further broken down by CAD-PRS distribution (top quintile and bottom 80%) (2). A minimum risk of 20% for developing CAD in 10 years was applied to employees classified as high risk by the PCE and 12.5% (average of moderate risk, 5 to < 20%) for those with moderate risk (9), respectively; a 1.9-fold increase in the risk was applied to employees in the top quintile of the CAD-PRS distribution (2). The risk of CAD increased among employees with ischemic stroke (0.017 [95% CI 0.014 – 0.019]) (12) and with statin-induced diabetes (HR: 2.270 [95% CI 1.950–2.650]) (13) but not for myopathy (14) or post-hemorrhagic stroke (HR, 1.600 [95% CI 0.300–2.900] (15).

**Table 1:** Annual parameter inputs.

**Domain**	**Description**	Baseline (range)^*^/[95% CI]	**Distributions Beta (α, β) Log normal (μ, σ) Gamma (α, β)**	**Sources**
Initial distribution	Low PCE risk	0.410 (0.205–0.615)	Beta (8.660, 12.460)	(2)
	Moderate PCE risk	0.363 (0.181–0.544)	Beta (9.450, 16.580)	(2)
	High PCE risk	0.227 (0.114–0.341)	Beta (11.610, 39.530)	(2)
Enrollment	Employee participation in WHP	0.520 (0.260–0.780)	Beta (6.860, 6.330)	(80)
Risk of CAD^†^	High PCE risk	0.022 (0.017–0.027)	Beta (76.390, 3380.450)	(9)
	Moderate PCE risk	0.013 (0.005–0.022)	Beta (9.330, 697.440)	(9)
	OR of CAD (high PRS)	1.900 (1.800–2.000)	Log normal (0.640, 0.050)	(2)
	HR of CAD (with diabetes)	2.000 [1.830–2.190]	Log normal (0.570, 0.220)	(13)
	CAD after ischemic stroke	0.017 (0.014–0.019)	Beta (174.590, 10095.920)	(12)
Ischemic Stroke	Risk ischemic stroke	0.004 (0.003–0.005)	Beta (95.650, 23817.310)	(81)
	Risk of ischemic stroke after CAD	0.015		(69)
	HR of ischemic stroke with diabetes	2.270 [1.950–2.650]	Log normal (0.370, 0.260)	(13)
	Risk of ischemic stroke post-hemorrhagic stroke	0.057 [0.048–0.068]	Beta (117.640, 1946.260)	(15)
Statin effectiveness	HR of CAD risk reduction	0.560 [0.400–0.780]	Log normal (−0.580, 0.090)	(17)
	HR for ischemic stroke risk reduction	0.770 [0.630–0.940]	Log normal (−0.260, 0.080)	(18)
Adherence	Statin adherence	0.500 (0.400–0.600)	Beta (47.520, 47.520)	Assumption (22, 23)
Statin side effects	Risk of myopathy	0.0001 (0.0001–0.0002)	Beta (2397.880, 4793360.990)	(25)
	Risk of diabetes	0.0015 (0.0010–0.0020)	Beta (847.590, 112165.190)	(25)
	Risk of hemorrhagic stroke	0.0002 (0.0001–0.0002)	Beta (862.670, 1149370.300)	(25)
Mortality^‡^	Risk of death, acute CAD	0.228 (0.182–0.274)	Beta (73.910, 250.270)	(26)
	Risk of death, post-acute CAD	0.070 (0.067–0.072)	Beta (14100.390, 58209.330)	(28)
	HR (diabetes and CAD)	1.810 [1.440–2.280]	Log normal (0.690, 0.090)	(30)
	Risk of death after ischemic stroke or hemorrhagic stroke and CAD	0.075 (0.050–0.100)	Beta (88.720, 1094.730)	Assumption (70)
	Risk of death, acute ischemic stroke	0.100 (0.080–0.120)	Beta (86.340, 777.020)	(27)
	Risk death, post-hemorrhagic or post-ischemic stroke	0.069 (0.055–0.082)	Beta (89.390, 1215.650)	(29)
	RR (with diabetes and ischemic stroke)	1.670 (1.580–1.760)	Log normal (0.800, 0.180)	(31)
	Risk, acute hemorrhagic stroke	0.390 (0.330–0.450)	Beta (98.620, 154.250)	(27)
	HR (diabetes versus no diabetes)	1.680 [1.520–1.870]	Log normal (0.510, 0.090)	(30)
Utility weights§	CAD	0.790 (0.730–0.860)	Beta (118.380, 31.46)0	(32)
	Myopathy	0.917 (0.896–0.938)	Beta (697.060, 54.950)	(34)
	Diabetes	0.800 (0.620–0.980)	Beta (14.380, 3.590)	(33)
	Stroke	0.630 (0.440–0.780)	Beta (18.890, 11.090)	(32)
Disutility weights	Acute CAD	0.041 (0.021–0.062)	Beta (14.690, 343.730)	(36)
	Acute stroke	0.220 [0.180–0.260]	Beta (90.420, 320.590)	(37)
	Age disutility	0.004 (0.002–0.006)	Beta (15.300, 3809.930)	(35)
Costs	CAD-PRS test	145 (116–174)	Gamma (96.040, 0.660)	Allelica, Inc
	Standard-WHP	58 (46–70)	Gamma (96.040, 1.660)	(82)
	Mobile health app	6 (5–7)	Gamma (96.040, 16.010)	Allelica, Inc
	Primary care visit	114 (91–137)	Gamma (96.040, 0.840)	(38)
	Statin therapy	132 (106–158)	Gamma (96.040, 0.730)	(83)
	Background healthcare costs	4,941 (3,953–5,930)	Gamma (96.040, 0.020)	(46)
**Acute**				
	Non-fatal CAD	65,442 (43,818–100,531)	Gamma (20.460, 0.0003)	(39)
	Fatal CAD	18,246 (14,597–21,896)	Gamma (96.040, 0.0053)	(40)
	Non-fatal ischemic stroke	40,225 (11,539–100,184)	Gamma (3.160, 0.0001)	(39)
	Fatal ischemic stroke	11,256 (9,005–13,507)	Gamma (96.040, 0.0085)	(40)
	Non-fatal hemorrhagic stroke	38,246 (30,596–45,895)	Gamma (96.040, 0.0025)	(71)
	Fatal hemorrhagic stroke	18,246 (14,597–21,896)	Gamma (96.040, 0.0053)	(40)
**Follow-up**				
	CAD	11,815 (7,865–16,186)	Gamma (30.990, 0.003)	(72)
	Stroke (hemorrhagic/ischemic)	20,005 (16,004 –24,006)	Gamma (96.040, 0.0048)	(41)
	Myopathy	20,438 (16,351–24,536)	Gamma (96.040, 0.0047)	(45)
	Diabetes	10,026 (8,021–12,031)	Gamma (96.040, 0.0096)	(44)
**Lost productivity**				
(Year of diagnosis)	CAD/stroke	73,492 (58,794–88,191)	Gamma (96.040–0.001)	(47, 48)
(Follow-up years)	CAD/stroke	9,056 (7,245–10,868)	Gamma (96.040–0.011)	(47, 73)
	Diabetes	9,242 (7,393–11,090)	Gamma (97.040–0.010)	(47, 49)
	Myopathy	9,056 (7,245–10,868)	Gamma (96.040–0.011)	(47, 73)

CAD, coronary artery disease; CI, confidence intervals; CAD-PRS, polygenic risk score for coronary artery disease; OR, odds ratio; HR, hazard ratio. ^*^Range (+/- 20% of the baseline value, except for enrollment and initial distribution parameters to account for a wide variation (+/- 50%) in the estimate). The range and 95% CI were used in the sensitivity analysis. ^†^Statin-induced myopathy did not change the risk of CAD and stroke or mortality (74–77). Further, the risk of CAD did not change after hemorrhagic stroke (15). ^‡^Mortality after stroke and CAD is significantly high compared to patients with only CAD or stroke. In a Medicare study population, more than 50% of patients with stroke and CAD died in the first year (70). Due to data limitations and our study population being younger compared to Medicare enrollment, we assumed 75% (50% - 100%) of patients will die in 10 years. We also assumed higher mortality for stroke among individuals with diabetes compared to those without diabetes based on a non-US study. Although no study has been carried out on the US population, studies including a meta-analysis showed increased mortality among stroke patients with diabetes (31, 78, 79). ^§^Due to the lack of data on the quality-of-life utilities among patients with multiple conditions, we assumed the lowest utility among the combination of diseases.

Although employees at risk of developing CAD are also likely to develop ischemic stroke (9), in this study, we assumed the risk of ischemic stroke to be equal to that of the general population. Around 800,000 cases of stroke occur among adults (around 200 million) in the United States per year and a significant majority (90%) are ischemic stroke cases (81). Based on these estimates, we derived a 0.004 annual risk of developing ischemic stroke, which we assumed to be constant over the time horizon of 5 years.

Statins are recommended as preventive therapy among individuals at high risk of CAD and have been shown to be effective (16) in reducing the risk of CAD (HR: 0.560 [95% CI 0.400–0.780]) (17) and stroke events (HR: 0.770 [95% CI 0.630–0.940]) (18). This risk reduction was only applied to employees that were adherent to the therapy. Simvastatin 20–80 mg is the most commonly used type of statin in the United States accounting for more than 42% of all prescriptions (19). We assumed the effectiveness of statins to be uniform across all eligible employees in the cohort although the efficacy of simvastatin among high- or moderate-risk individuals with high CAD-PRS has not been examined. Therefore, we used the efficacy of pravastatin, which has been examined among high-risk individuals with high CAD-PRS (17). Further, pravastatin and simvastatin have shown comparable effectiveness in reducing LDL cholesterol (20, 21).

Half of the employees identified as eligible for preventive therapy were assumed to be adherent to statin over the analytical time horizon. Although low (< 50%) adherence to prevention therapy has been reported in the literature (22, 23), no study has examined adherence to statins among individuals with high CAD-PRS. Despite this gap in the literature, there is evidence of higher adherence to preventive therapy among women with high breast cancer PRS (24). Those adherent to statin preventive therapy have an additional annual risk of developing adverse effects: myopathy (0.010%), diabetes (0.150%), and hemorrhagic stroke (0.020%) (25). Those with diabetes have an increased risk of CAD (HR: 2.000 [1.830–2.190]) (13) and ischemic stroke (HR: 2.270 [1.950–2.650]) (13), and those with hemorrhagic stroke have a higher risk of ischemic stroke (0.057 [0.048–0.068]) (15).

The risk of death among event-free employees was based on the social security life tables (Supplementary Table S2). Acute coronary syndrome (0.228 [0.182–0.274]) (26), ischemic stroke (0.100 [0.080–0.120) (27), and hemorrhagic stroke (0.390 [0.330–0.450]) (27) had a higher risk of death compared to chronic CAD (0.070 [0.067–0.072]) (28) and ischemic and hemorrhagic stroke (0.069 [0.055–0.082]) (29), respectively. Diabetes significantly increased the risk of death by nearly 2-fold among employees with CAD (HR: 1.810 [1.440–2.280]) (30) and ischemic stroke (HR: 1.670 [1.580–1.760]) (31) and compared disease-free employees (HR: 1.680 [1.520–1.870]) (30).

### Utility values

Utility weights were assigned to health states in the model to reflect their health status (severity of disease) with death assigned a utility weight of 0 and event-free health states assigned a utility weight of 1 (perfect health). Utility weights for CAD (0.790, 0.730–0.860) (32), ischemic and hemorrhagic stroke (0.640, 0.440–0.780) (32), diabetes (0.800, 0.620–0.980) (33), and myopathy (0.917, 0.896–0.938) (34) were derived from the literature. An annual decrement was applied to reflect disutility due to aging (35) and disutility for acute events [CAD (36) and ischemic and hemorrhagic stroke (37)].

### Costs

Costs came from the literature and were inflation adjusted to US$ 2019 using the gross domestic product deflator. We considered both medical costs (PCE screening test, CAD-PRS testing, statin preventive therapy, primary care visit, and disease treatment costs) and lost productivity (absenteeism and presenteeism). The cost of CAD-PRS testing ($145) was based on current prices of genotyping arrays and bioinformatic analysis needed to develop CAD-PRS (Source: Allelica, Inc) while the cost of Standard-WHP ($58) came from gray literature and reflected the average cost per employee to perform workplace biometric screening (82). CAD-PRS is a one-time cost and was only applied in the first year and PCE is performed annually. Therefore, starting from the second year, both the CardioriskSCORE and Standard-WHP strategies assessed the risk of CAD using PCE and had the same annual cost. For the CardioriskSCORE strategy, an additional $6 for the Health Assistant Mobile App was applied after the first year, and a one-time primary care visit cost ($114) for genetic consultation, based on a nationally presentative medical expenditure panel survey on patient medical expenses (38), was applied to the cohort with high CAD-PRS. The cost of statin preventive therapy ($132) was derived from online pharmacy prices and applied only to employees that were adherent to the therapy (83).

The cost of treating acute and chronic CAD ($65,442; $11,815) and ischemic stroke ($40,225; $20,005) was based on costs for patients with cardiovascular diseases in the United States (39–41), respectively. The cost of recurrent CAD (42) and ischemic stroke (43) was calculated as the product of the risk of recurrence of the event and the cost of treatment. Costs of treating diabetes ($10,026) (44), myopathy ($20,438) (45), and hemorrhagic stroke ($20,005) (41) were included for employees that experienced statin preventive therapy side effects. Background healthcare costs were applied to all individuals that are alive to account for healthcare resource utilization, which was estimated based on per capita healthcare expenditure for privately insured individuals in the United States (46).

The illness of employees has a substantial impact on productivity and employer expenses. Therefore, we considered health-related absenteeism (time taken off work due to illness) and presenteeism (reduction in productivity at work due to illness) as lost productivity from an employer's perspective. Employees that survived CAD, ischemic, and hemorrhagic stroke had a total loss in productivity of $73,492 during their first year of diagnosis (47, 48) and $9,056 in subsequent years (47, 73). We estimated the cost of absenteeism based on patients who survived acute coronary artery syndromes and had an average absenteeism cost of $14,698 in their first year (48). To our knowledge, no study has examined absenteeism for CAD and stroke after the first year of diagnosis, and for myopathy. Therefore, we assumed an absenteeism cost of $1,811 for myopathy, CAD, and stroke in subsequent years, which is equivalent to the national average cost of absenteeism for people living with chronic conditions (73). The cost of presenteeism was assumed to be four times the cost of absenteeism, derived from the national estimates on lost productivity costs incurred by the employer due to coronary heart disease and stroke (47). Annual lost productivity cost for employees living with diabetes was $9,242, which includes both absenteeism and presenteeism costs (47, 49). For consistency, we also assumed presenteeism costs for myopathy and diabetes to be four times the cost of absenteeism.

### Analysis

The analysis was performed to reflect a self-insured employer perspective. In the United States, employers who are self-insured bear the cost burden of medical claims resulting from adverse outcomes among insured employees. This perspective was chosen to examine the cost implications to the employer and the health benefits of preventing cardiovascular adverse events among employees. The time horizon for the analysis was 5 years, which represented the average number of years an employee consistently works with a particular employer in the United States (84).

The main outcomes were total costs, incremental costs, incremental quality-adjusted life years (QALYS), and incremental cost-effectiveness ratio (ICER). We assessed the relative Performance of the three strategies using the ICER. A strategy was considered cost-effective compared to the second best alternative if the ICER < willingness-to-pay (WTP) threshold of $50,000. We discounted future costs and QALYs at the same rate of 3% per year as recommended for cost-effectiveness analysis of interventions in healthcare in the United States (50).

One-way and probabilistic sensitivity analyses were used to assess uncertainty in model parameters and their impact on the incremental net monetary benefit. In one-way sensitivity analysis, we varied one parameter at a time while holding other parameters at baseline value and results are presented using a tornado diagram. In probabilistic sensitivity analysis, we assigned beta (probability parameters), log-normal (relative risk and hazard ratio parameters), and gamma (costs) distributions and performed 10,000 Monte Carlo simulations. Results are reported using a joint distribution of incremental costs and QALYs gained for the CardioriskSCORE vs. Standard-WHP and CardioriskSCORE vs. No-WHP.

### Scenario analysis

In the scenario analysis, we accounted for annual changes in the risk of CAD with the movement of fractions of the cohort across risk categories (low, moderate, and high) by strategy: with a WHP (CardioriskSCORE and Standard-WHP) and without a WHP (No-WHP) (51). Per model cycle, fractions of the cohort can move from low to moderate, low to high, moderate to low, moderate to high, high to low, and high to moderate. We assumed that the cohort in the No-WHP strategy experience a natural change in the risk CAD (25, 5, 35, 20, 6, and 31%) based on the study that reported annual changes in cardiovascular risk (51).

In the CardioriskSCORE and Standard-WHP, the change in risk was weighted to account for the percentage of the cohort that enrolled in a WHP. The fraction of the cohort that did not enroll in a WHP (48%) experienced the natural change in risk while those that enrolled (52%) had changes in CAD risk that is comparable to that of individuals in a WHP (12.050, 0.620, 46.350, 7.660, 15.650, and 48.700%). Based on the percentage enrollment, the change in CAD risk weighed was 18.780, 2.900, 40.450, 14.080, 10.630, and 39.500%. We applied for annual PCE screening and assumed that enrollment remained constant.

As polygenic screening is only performed once in the first year of the time horizon, follow-up costs per patient screened are similar for CardioriskSCORE and Standard-WHP. Changes in CAD risk occurred after the first year.

## Results

**Figure 2:**
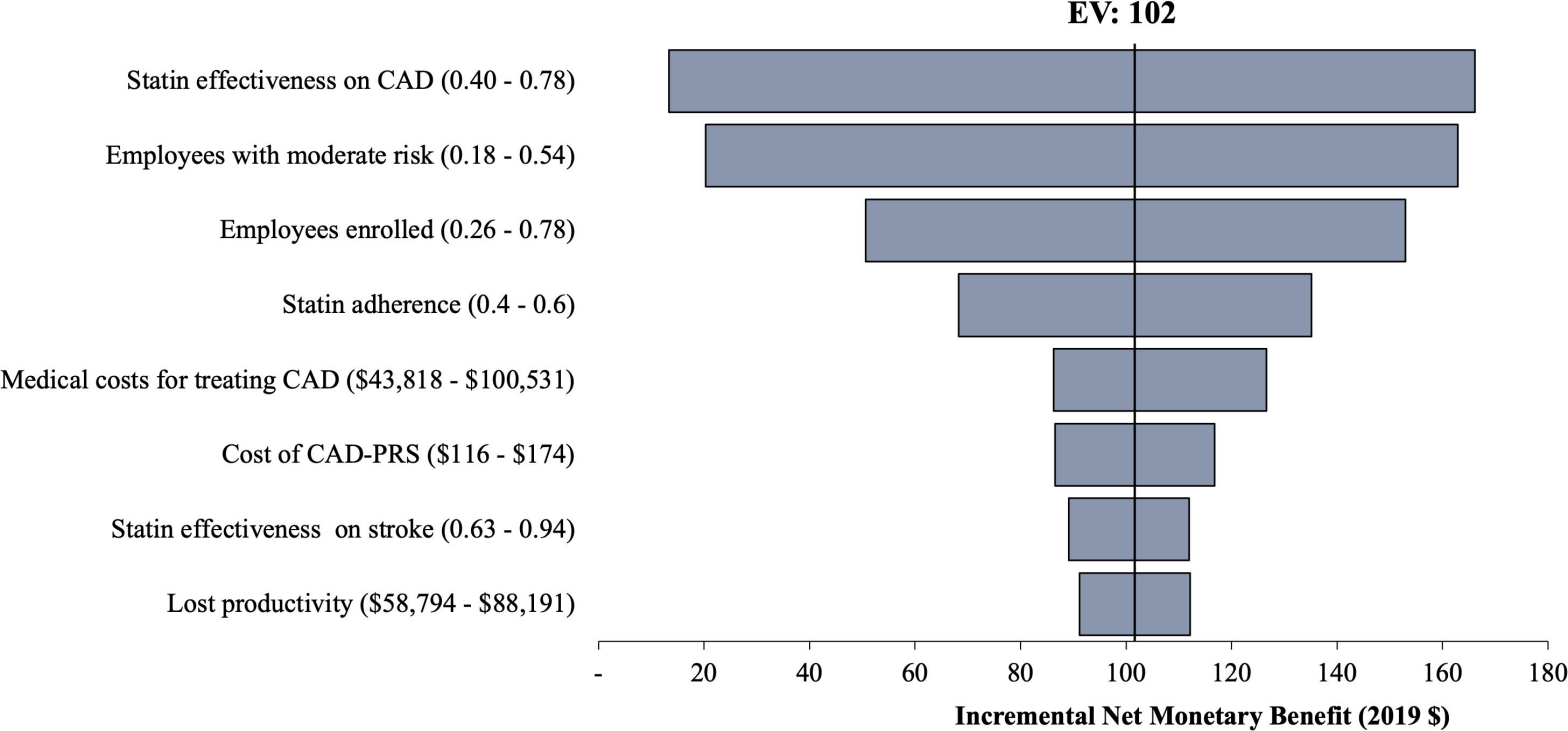
One-way sensitivity analysis of CardioriskSCORE compared to Standard-WHP. This figure shows results from the one-way sensitivity analysis for CardioriskSCORE compared to the Standard-WHP. Variation in parameter values did not change conclusions in the base case findings as indicated with positive incremental net monetary benefit across all parameters. Stain effectiveness on CAD had the highest impact on the findings. CAD, coronary artery disease; CAD-PRS, polygenic risk score for coronary artery disease; EV, expected value.

**Figure 3:**
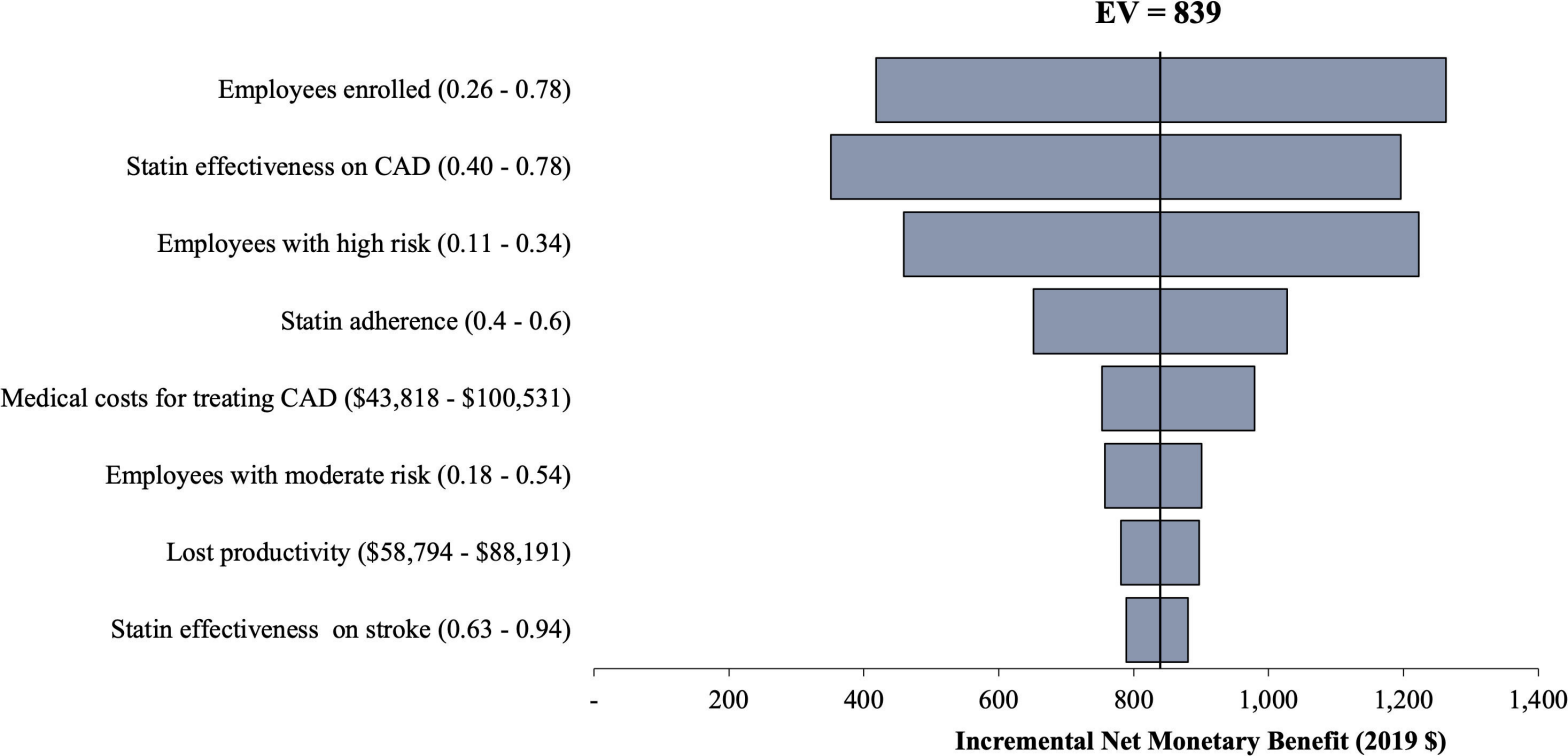
One-way sensitivity analysis of CardioriskSCORE compared to No-WHP. This figure shows results from the one-way sensitivity analysis for CardioriskSCORE compared to No-WHP. Base case results remain robust to variations in parameter values as indicated with positive incremental net monetary benefit across all parameters. Similar to the Standard-WHP strategy, stain effectiveness on CAD had the highest impact on the findings. CAD, coronary artery disease; No-WHP, no-workplace health program; EV, expected value.

Base case analysis results are reported in Table 2. CardioriskSCORE was a dominant strategy with lower incremental cost ($53 and $575) and higher QALYs (0.001 and 0.005) per employee compared to Standard-WHP and No-WHP, respectively. Findings remained robust in the one-way sensitivity analysis (Figures 2, 3). CardioriskSCORE had an incremental net monetary benefit of $102 and $839 compared to Standard-WHP and No-WHP, respectively. All parameter variations had a positive incremental net monetary benefit, indicating that CardioriskSCORE was dominant despite changes in parameter values. Statin effectiveness, baseline PCE risk among employees, the proportion of employees enrolled in the program, and statin adherence were the main parameter inputs impacting the findings.

**Table 2:** Results for the base case analysis in 5 years.

**Strategy**	Costs (US$)^*^	**Incremental costs**	QALYs^*^	**QALYs gained**	**ICER**
CardioriskSCORE	29,668 24,197–35,591	-	4.507 4.435 –4.578	-	Dominant
Standard-WHP	29,722 24,222–35,669	53	4.506 4.434 –4.577	−0.001	-
No-WHP	30,243 24,561–36,412	575	4.502 4.429 –4.574	−0.005	-

PCE, Pooled cohort equation; PRS, polygenic risk score; QALYs, quality-adjusted life years; ICER, incremental cost-effectiveness ratio; CAD, coronary artery disease; No-WHP, no wellness health program. ^*^Intervals represent 2.5th to 97.5th percentiles. This table shows base case cost-effectiveness analysis results for a 5-year time horizon in a cohort of 50-year-old employees. Compared to Standard-WHP and No-WHP alternatives, CardioriskSCORE had higher mean QALYs (0.001, 0.005) and lower mean costs ($53, $575) per employee.

Probabilistic sensitivity analysis results in Supplementary Figures S1, S2 underscore the robustness of the findings in the base case analysis. The joint distribution of incremental costs and incremental effectiveness (QALYs gained) of CardioriskSCORE compared to Standard-WHP and No-WHP indicate a higher likelihood of cost-saving and improved quality of life among employees. At a $50,000 willingness-to-pay threshold, CardioriskSCORE had a 95 and 99% probability of being cost-effective compared to Standard-WHP and No-WHP, respectively.

Results from the scenario analysis (Table 3) were consistent with base case results. After adjusting for changes in the annual risk of CAD CardioriskSCORE remained cost-saving and effective with higher mean QALYs (0.001, 0.009) and lower mean costs ($28, $1,306) per employee in the primary prevention population. In the scenario analysis, a larger fraction of the cohort moved to the low-risk category in the CardioriskSCORE due to the impact of the program and compared to No-WHP, which resulted in more cost-savings and QALYs gained ($1,306; 0.009) than in the base case analysis ($575; 0.005).

**Table 3:** Cost-effectiveness results after accounting for changes in CAD risk.

**Strategy**	Costs (US$)^*^	**Incremental costs**	QALYs^*^	**QALYs gained**	**ICER**
CardioriskSCORE	26,722 22,097–31,718	-	4.537 4.466–4.604	-	Dominant
PCE-alone	26,750 22,105–31,754	28	4.536 4.466–4.603	−0.001	-
No-WHP	28,028 23,194–33,229	1,306	4.528 4.457–4.596	−0.009	-

PCE, Pooled cohort equation; PRS, polygenic risk score; QALYs, quality-adjusted life years; ICER, incremental cost-effectiveness ratio; CAD, coronary artery disease; No-WHP, no wellness health program. ^*^Intervals represent 2.5th to 97.5th percentiles. This table shows cost-effectiveness analysis results after adjusting for changes in the risk of CAD per year within a 5-year time horizon in a cohort of 50-year-old employees. CardioriskSCORE had higher mean QALYs (0.001, 0.009) and lower mean costs ($28, $1,306) per employee in the primary prevention population, which is consistent with the base case analysis.

## Discussion

This study found that integrating a CAD-PRS in a workplace cardiovascular disease prevention program is cost-effective and cost-saving with more than $53 and $575 per employee screened, compared to Standard-WHP and No-WHP, respectively. These findings were robust to variations in parameter values and annual changes in the risk of CAD.

The findings in the current study are broadly consistent with the literature. WHPs focused on healthy lifestyles, physical activity, and nutrition were found to be cost-effective with an increase in QALYs (0.003) (52), which is comparable to QALYs gained by the CardioriskSCORE. Furthermore, WHPs were found to be cost-saving with a reduction in medical expenses and absenteeism of $3.270 and $2.730 per dollar spent, respectively (53). In a cost-effectiveness microsimulation model on the ban of sugar-sweetened beverages at a workplace, the intervention was found to be effective at reducing the incidence of chronic conditions, medical costs, and lost productivity, by saving $300,000 per 10,000 employees over a period of 10 years (54).

Prior studies focused on cardiovascular disease prevention in workplace settings have shown positive results, but few examined the costs and cost-effectiveness of programs, and none accounted for genetic risk. Cardiovascular disease prevention programs were found to improve the awareness of risk factors among firefighters (55) early detection of isolated risk factors (56), healthy lifestyle (57) and reduced obesity, high blood pressure, and hyperlipidemia (58). A lifestyle education program focused on cardiovascular disease risk reduction within a period of 12 months was found cost-effective with a $454 per percentage point reduction in the Framingham Risk Score for coronary heart disease risk (59). Among firefighters, a cardiovascular prevention program was cost-effective compared to doing nothing by preventing 10% of cardiovascular events at $1,440 over 10 years (60).

Our findings are novel and address a key gap in the workplace health literature by demonstrating added economic value and improved quality of life of polygenic testing. Multiple vendors currently offer employer-based genetic testing in the United States with over 70% reporting employer cost reduction and improved employee health outcomes but there is no evidence of cost-effectiveness for their products (6). Overall, there is limited literature on precision medicine in workplaces, but prior work has explored the use of genetic testing and personalized interventions to improve employee health outcomes, protect workers at high risk, and reduce costs for workers' compensation (61). Furthermore, a review of the literature from the National Institute of Occupational Safety and Health (NIOSH) found that evidence on genetic testing in workplaces is limited and genetic tests for monitoring or screening need to be validated to provide reliable exposure or risk assessments (62). This shows that more research is needed to better understand the utility of precision medicine in workplaces, practical approaches to implementation, and employee data privacy.

In 2011, NIOSH launched the “Total Worker Health” program which defines company policies, programs, and practices that integrate protection from work-related safety and health hazards with the promotion of injury and illness-prevention efforts to advance worker wellbeing (63). Workplace health promotion programs that incorporate the Total Worker Health approach are especially necessary for addressing challenges faced by employees with long-term effects of COVID-19 (64) and have also been found to have a high return on investment (65). An indication that with the integration of PRS in programs that use the Total Worker Health approach, employers may gain an even higher return on investment from the added value in productivity and cost-savings from early disease detection and prevention.

Over 95% of employers in the United States offer some form of WHPs to identify health risks and manage chronic conditions (1). However, less than half focus on cardiovascular disease prevention (1), despite high disease prevalence in the workforce and costing employers more than $363 billion in medical expenses and causing productivity loss annually (66). Even more concerning is that cardiovascular deaths increased during the COVID-19 pandemic (67). Adding genetic testing in workplaces would provide value for employees by knowing their genetic risk and potentially changing their lifestyles to mitigate the risk of disease and associated healthcare costs and for employers by saving costs on employee medical claims and improving employee productivity (7).

This study has several limitations. First, in the base case analysis, the risk of CAD was assumed to be constant although it may change over time due to changes in behaviors and lifestyle. However, in this scenario, we accounted for the change in CAD risk and the results remained robust. Second, the model assumes that employees' risk can only be determined through workplace programs, which may not always be the case. Third, there could be a correlation across parameters that may impact the findings, which we did not account for in the model. Fourth, although CAD-PRS was estimated using a large multi-centric multi-ancestry population, the model in this study did not account for racial composition in workplace settings, which is a key predictor of cardiovascular outcomes. Finally, the model assumed that employees who agree to participate in the PCE risk assessment would also agree to PRS testing although there are privacy concerns regarding genetic testing in workplaces. However, the majority of employees have shown a willingness to perform genetic testing if the test is easy and accessible and the data are only available to employees and their doctors (7).

Adding polygenic screening in cardiovascular disease prevention saves costs for the employer and improves the quality of life of employees. The current COVID-19 pandemic has transformed the traditional workplace environment with more Americans working remotely and for longer hours with less access to gyms and other amenities for physical activity leading to an increased risk of cardiovascular diseases (68). There is an urgent need for innovative WHPs focusing on cardiovascular disease prevention and management to reduce future medical expenses and improve employees' health status and productivity.

## Data availability statement

The original contributions presented in the study are included in the article/Supplementary material, further inquiries can be directed to the corresponding author.

## Author contributions

DM developed the Markov model, performed the analysis, and drafted the manuscript. GB contributed to the conceptual development of the Markov model and writing the manuscript. JK, PD, and GBB contributed to the writing of the manuscript. All authors reviewed and approved the manuscript.
