# Supplementary material for: Cost-effectiveness analysis of implementing polygenic risk score in a workplace cardiovascular disease prevention program
Source: Front Public Health. 2023 Jul 11;11:1139496. doi: 10.3389/fpubh.2023.1139496 (PMC10366377; doi:10.3389/fpubh.2023.1139496)
Supplement: Supplementary file 1 [file Data_Sheet_1.docx]

***Supplemental Material***

**Title**

Cost-effectiveness analysis of implementing polygenic risk score in a workplace cardiovascular disease prevention program.

Deo Mujwara, PhD^1^; Jen Kintzle, MS^1^, Paolo Di Domenico, BSc^1^﻿; George B. Busby, DPhil^1^; Giordano Bottà, PhD﻿^1*^

^1^Allelica, Inc. New York, NY, USA.

Correspondence: Giordano Bottà (contact: [giordano@allelica.com](mailto:giordano@allelica.com))

**Table S1: Consolidated Health Economic Evaluation Reporting Standards (CHEERS) 2022 Checklist**

| **Topic** | **No.** | **Item** | **Location where item is reported** |
| --- | --- | --- | --- |
| Title |  |  |  |
|  | 1 | Identify the study as an economic evaluation and specify the interventions being compared. | Title page |
| Abstract |  |  |  |
|  | 2 | Provide a structured summary that highlights context, key methods, results, and alternative analyses. | Abstract page |
| Introduction |  |  |  |
| Background and objectives | 3 | Give the context for the study, the study question, and its practical relevance for decision making in policy or practice. | Introduction, pages 1 and 2 |
| Methods |  |  |  |
| Health economic analysis plan | 4 | Indicate whether a health economic analysis plan was developed and where available. | Not Applicable |
| Study population | 5 | Describe characteristics of the study population (such as age range, demographics, socioeconomic, or clinical characteristics). | Study population, page 2 |
| Setting and location | 6 | Provide relevant contextual information that may influence findings. | Study population, page 2 |
| Comparators | 7 | Describe the interventions or strategies being compared and why chosen. | Strategies, pages 2 and 3 |
| Perspective | 8 | State the perspective(s) adopted by the study and why chosen. | Analysis, page 9 (first paragraph) |
| Time horizon | 9 | State the time horizon for the study and why appropriate. | Analysis, page 9 (first paragraph) |
| Discount rate | 10 | Report the discount rate(s) and reason chosen. | Analysis, page 9 (second paragraph) |
| Selection of outcomes | 11 | Describe what outcomes were used as the measure(s) of benefit(s) and harm(s). | Analysis, page 9 (second paragraph) |
| Measurement of outcomes | 12 | Describe how outcomes used to capture benefit(s) and harm(s) were measured. | Analysis, page 9 (second paragraph) |
| Valuation of outcomes | 13 | Describe the population and methods used to measure and value outcomes. | Analysis, page 9 (second paragraph) |
| Measurement and valuation of resources and costs | 14 | Describe how costs were valued. | Parameter inputs, pages 4-8 |
| Currency, price date, and conversion | 15 | Report the dates of the estimated resource quantities and unit costs, plus the currency and year of conversion. | Costs, page 6 (paragraph 3) |
| Rationale and description of model | 16 | If modelling is used, describe in detail and why used. Report if the model is publicly available and where it can be accessed. | Model structure, page 3 |
| Analytics and assumptions | 17 | Describe any methods for analysing or statistically transforming data, any extrapolation methods, and approaches for validating any model used. | Model structure, page 3 |
| Characterising heterogeneity | 18 | Describe any methods used for estimating how the results of the study vary for subgroups. | Not Applicable |
| Characterising distributional effects | 19 | Describe how impacts are distributed across different individuals or adjustments made to reflect priority populations. | Not Applicable |
| Characterising uncertainty | 20 | Describe methods to characterise any sources of uncertainty in the analysis. | Analysis, page 9 (paragraph 3); scenario analysis, page 10 |
| Approach to engagement with patients and others affected by the study | 21 | Describe any approaches to engage patients or service recipients, the general public, communities, or stakeholders (such as clinicians or payers) in the design of the study. | Not Applicable |
| Results |  |  |  |
| Study parameters | 22 | Report all analytic inputs (such as values, ranges, references) including uncertainty or distributional assumptions. | Table 1, pages 20-23 |
| Summary of main results | 23 | Report the mean values for the main categories of costs and outcomes of interest and summarise them in the most appropriate overall measure. | Results, page 11 (paragraph 1) |
| Effect of uncertainty | 24 | Describe how uncertainty about analytic judgments, inputs, or projections affect findings. Report the effect of choice of discount rate and time horizon, if applicable. | Results, pages 11 and 12 (paragraph 2 and 3) |
| Effect of engagement with patients and others affected by the study | 25 | Report on any difference patient/service recipient, general public, community, or stakeholder involvement made to the approach or findings of the study | Not Applicable |
| Discussion |  |  |  |
| Study findings, limitations, generalisability, and current knowledge | 26 | Report key findings, limitations, ethical or equity considerations not captured, and how these could affect patients, policy, or practice. | Discussion, pages 11-13 |
| Other relevant information |  |  |  |
| Source of funding | 27 | Describe how the study was funded and any role of the funder in the identification, design, conduct, and reporting of the analysis | Funding statement |
| Conflicts of interest | 28 | Report authors conflicts of interest according to journal or International Committee of Medical Journal Editors requirements. | Conflict of interest statement |

### **Table S2: Age specific annual probability of death**

| **Age** | **Baseline value** | **Lower bound value** | **Upper bound value** |
| --- | --- | --- | --- |
| 40 | 0.0020 | 0.0015 | 0.0024 |
| 41 | 0.0021 | 0.0015 | 0.0026 |
| 42 | 0.0022 | 0.0016 | 0.0027 |
| 43 | 0.0023 | 0.0017 | 0.0029 |
| 44 | 0.0025 | 0.0019 | 0.0031 |
| 45 | 0.0027 | 0.0020 | 0.0033 |
| 46 | 0.0029 | 0.0022 | 0.0036 |
| 47 | 0.0031 | 0.0023 | 0.0039 |
| 48 | 0.0034 | 0.0025 | 0.0042 |
| 49 | 0.0037 | 0.0028 | 0.0046 |
| 50 | 0.0041 | 0.0030 | 0.0051 |
| 51 | 0.0044 | 0.0033 | 0.0055 |
| 52 | 0.0048 | 0.0036 | 0.0061 |
| 53 | 0.0053 | 0.0040 | 0.0066 |
| 54 | 0.0058 | 0.0043 | 0.0072 |
| 55 | 0.0063 | 0.0047 | 0.0079 |
| 56 | 0.0068 | 0.0051 | 0.0085 |
| 57 | 0.0074 | 0.0056 | 0.0092 |
| 58 | 0.0080 | 0.0060 | 0.0100 |
| 59 | 0.0086 | 0.0064 | 0.0107 |
| 60 | 0.0092 | 0.0069 | 0.0115 |
| 61 | 0.0099 | 0.0074 | 0.0124 |
| 62 | 0.0106 | 0.0080 | 0.0133 |
| 63 | 0.0113 | 0.0085 | 0.0142 |
| 64 | 0.0121 | 0.0091 | 0.0151 |
| 65 | 0.0129 | 0.0097 | 0.0162 |
| 66 | 0.0139 | 0.0105 | 0.0174 |
| 67 | 0.0150 | 0.0113 | 0.0187 |
| 68 | 0.0162 | 0.0122 | 0.0202 |
| 69 | 0.0175 | 0.0132 | 0.0219 |
| 70 | 0.0191 | 0.0144 | 0.0238 |
| 71 | 0.0209 | 0.0157 | 0.0260 |
| 72 | 0.0229 | 0.0172 | 0.0285 |
| 73 | 0.0250 | 0.0188 | 0.0312 |
| 74 | 0.0275 | 0.0207 | 0.0342 |
| 75 | 0.0303 | 0.0228 | 0.0377 |
| 76 | 0.0336 | 0.0253 | 0.0418 |
| 77 | 0.0372 | 0.0280 | 0.0463 |
| 78 | 0.0411 | 0.0310 | 0.0511 |
| 79 | 0.0455 | 0.0343 | 0.0565 |
| 80 | 0.0505 | 0.0381 | 0.0627 |
| 81 | 0.0563 | 0.0425 | 0.0699 |
| 82 | 0.0627 | 0.0474 | 0.0778 |
| 83 | 0.0697 | 0.0527 | 0.0863 |
| 84 | 0.0774 | 0.0587 | 0.0958 |
| 85 | 0.0861 | 0.0653 | 0.1065 |
| 86 | 0.0960 | 0.0729 | 0.1185 |
| 87 | 0.1071 | 0.0815 | 0.1321 |
| 88 | 0.1196 | 0.0911 | 0.1472 |
| 89 | 0.1334 | 0.1018 | 0.1639 |
| 90 | 0.1486 | 0.1137 | 0.1822 |
| 91 | 0.1650 | 0.1265 | 0.2019 |
| 92 | 0.1827 | 0.1404 | 0.2229 |
| 93 | 0.2016 | 0.1554 | 0.2453 |
| 94 | 0.2216 | 0.1713 | 0.2689 |
| 95 | 0.2416 | 0.1873 | 0.2923 |
| 96 | 0.2613 | 0.2032 | 0.3151 |
| 97 | 0.2802 | 0.2185 | 0.3370 |
| 98 | 0.2980 | 0.2331 | 0.3574 |
| 99 | 0.3142 | 0.2464 | 0.3759 |
| 100 | 0.3314 | 0.2606 | 0.3954 |

**Figure S1: incremental cost-effectiveness for CardioriskSCORE versus Standard-WHP**


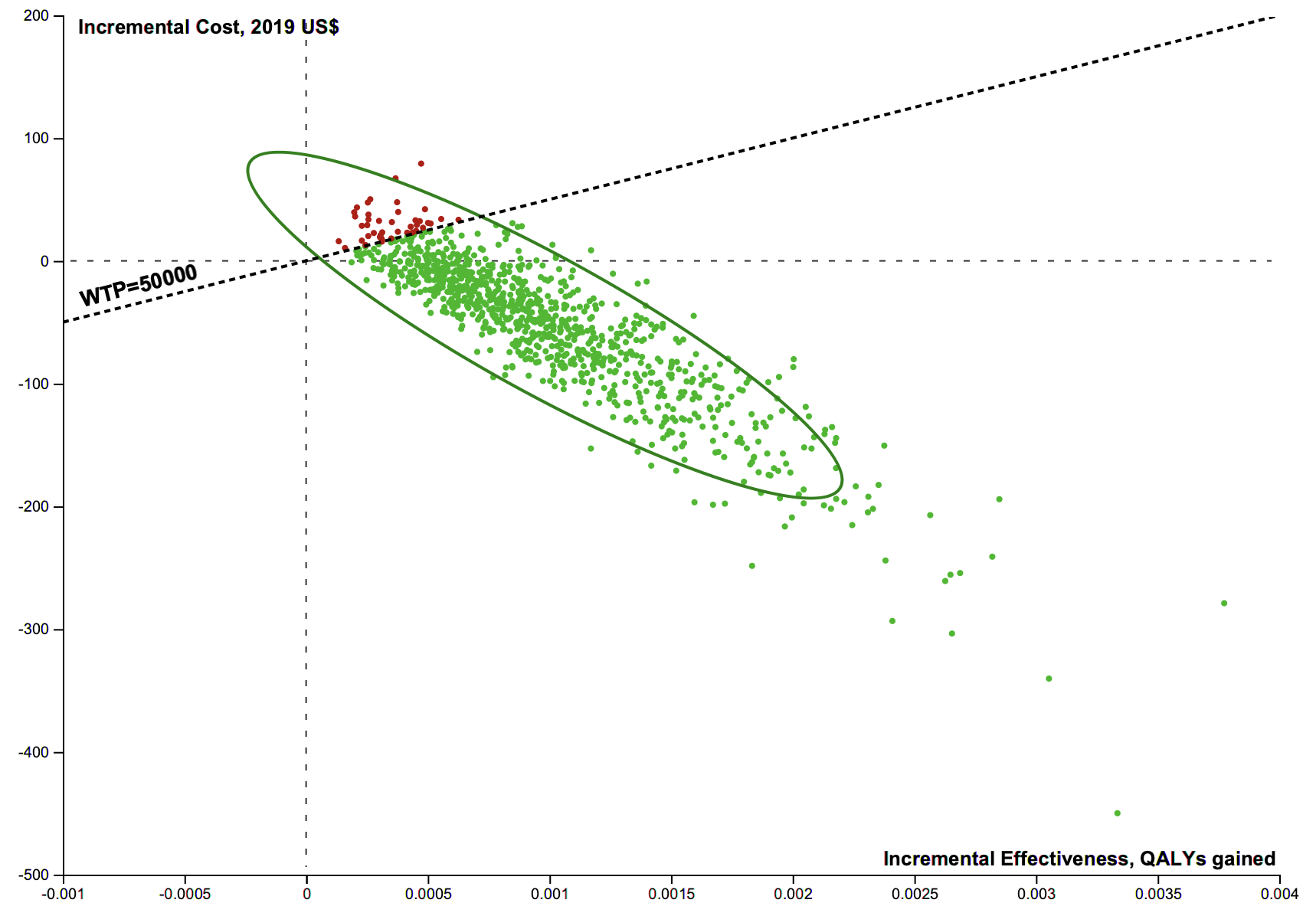


Abbreviations: PCE = Pooled cohort equation; QALYs = Quality adjusted life years; WTP = willingness to pay; WHP=Wellness health program

Figure S1 shows the joint distribution of the incremental cost and incremental effectiveness of the CardioriskSCORE program compared with PCE-Alone. A significant majority (95%) of the distribution falls below the WTP, an indication that CardioriskSCORE is more likely to be cost-effective compared to PCE-Alone.

**Figure S2: incremental cost-effectiveness for CardioriskSCORE versus No-WHP**


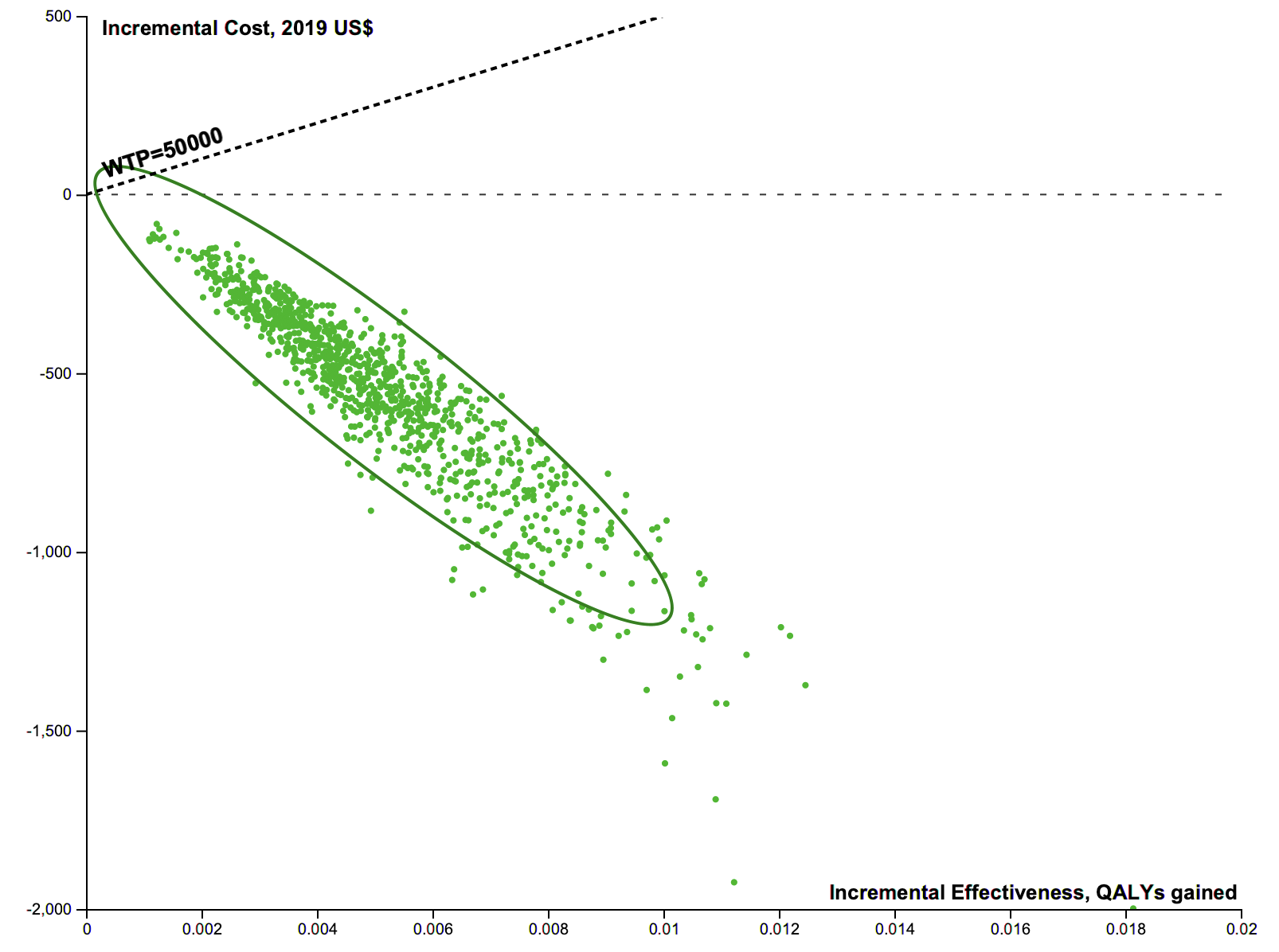


Abbreviations: No-WHP = No wellness health program; QALYs = Quality adjusted life years; WTP = willingness to pay

Figure S2 shows the joint distribution of the incremental cost and incremental effectiveness of CardioriskSCORE compared with No-WHP. Nearly all of the distribution falls below the WTP, an indication that CardioriskSCORE nearly cost-effective all the time when compared to No-WHP.
